# Selections and Abstracts

**Published:** 1915-07

**Authors:** 


### SELECTIONS AND ABSTRACTS

### THE CAMPAIGN AGAINST CANCER.

SYMPOSIUM: LOOKING TO THE INCREASED KNOWLEDGE OF CANCER.

---

### EDUCATION OF THE PUBLIC CONCERNING CANCER OF THE UTERUS.

By JOHN G. CLARK, M. D., PHILADELPHIA.

(Continued from Last Number)

The time of consultation after recognition of the first signs is noted in parallel columns:

| | | |
|---|---|---|
| Within first month | 14 per cent | 22 per cent. |
| After first month | 18 per cent | 35 per cent. |
| After third month | 26 per cent | 33 per cent. |
| After sixth month | 12 per cent | 5 per cent. |
| After ninth month | 18 per cent | 5 per cent. |
| After twelfth month | 12 per cent | 0 per cent. |

Failure to consult a physician was ascribed by the patients to the following causes:

| | |
|---|---|
| Failure to recognize danger of symptoms | 30 |
| Carelessness in consulting a physician | 19 |
| Modesty | 4 |
| Fear of consultation | 14 |
| Inability to consult physician on account of poverty | 2 |

As the advice was sought earlier so was it more promptly accepted. Thus from 1898 to 1902 only seventy-eight per cent were operated upon within fourteen days after advice was given; while in 1903 the percentage had risen to ninety.

As a result of the study by the excellent statistician and investigator, Sampson, of 412 cases of cancer admitted to the Johns Hopkins Hospital, the following results were recorded: Bleeding in some form or other was noted in ninety-three per cent of the patients. It varied from a slight degree ("only a show"), appearing at irregular intervals, as on exertion, after coitus, using a douche, or straining at stool. In some cases it was slight but constant, as indicated by soiled linen worn during the day. In other instances the flow was more profuse, appearing like a prolonged or irregular menstruation, or, in women after the menopause, like a return of the period. Severe hemorrhage marked still another initial symptom.

From this study it is evident that chief stress must be laid upon menstrual deviations in reaching an early diagnosis. In this disease days are vital to the patient's welfare, for the course of cancer is usually rapid. Patients rarely live over three years; three-fourths succumb within two, and one-third within one year after the first manifestation of the disease. As Sampson so clearly emphasizes, a very short period of neglected bleeding determines the horrible fate of the patient. In our country only thirty per cent of cases are operable when diagnosticated, and only ten to twenty-five per cent of such cases remain free from recurrence after five years.

As guardians of the health let us first imprint deeply upon our consciences the extreme hazard and awful responsibility of procrastination in making an immediate examination and setting at rest this very important question. Culpable indeed is the physician who delays in such cases. With the best foresight errors of commission as well as of omission in medicine are unavoidable, but the error of omission in this class of cases will soon be regarded by the laity as criminal an oversight as permitting an acute appendicitis to pass from an operable stage over into a lethal peritonitis before it is recognized.

Carcinoma Age. Age as a factor should also have its fullest qualifications. The young woman should be taught that irregularities of menstruation are abnormal, but that they seldom, if ever, indicate malignant trouble. On the contrary women over thirty-five, and particularly between forty and forty-five years of age, with such signs are in danger and should not rest until a diagnosis is reached by some competent physician. The instructive scheme, drawn from Kroemer's statistics, and shown on this page, has been constructed to indicate more vividly the carcinoma periods in a woman's life.

HOW MAY THE CHARLATAN'S END BE CIRCUMVENTED?

While with our profession it is a well-established axiom that early operation is the only cure for cancer, to the laymen this remedy is the one most abhorred. The terror inspired by the knife is so well known that every vampire seeks to offset this horror by advertising in the most seducive way a sure cure without surgical means. Through the courtesy of Dr. Green, the assistant secretary of the American Medical Association, I have received scores of clippings of such advertisements from the most reputable journals and newspapers in the United States, and in some instances these advertisements have been further bolstered up by commendatory editorials. Prominent headlines are devoted to the statement that cancer is not cured by the knife—a statement which appears glaringly patent to every layman when he recalls the number of individuals, probably within his knowledge, who have not been relieved by an operation or have succumbed immediately from its effects. In surgical cases the layman hears of the mishaps and fatalities, while the good results may be overlooked.

In patients who have been relieved of cancer by operation, the knowledge of the exact nature of the disease is usually withheld from them. In the one fatally stricken by this malady the hallmarks are too clear to be disguised. This is, therefore, a well-founded opinion among laymen that cancer is incurable by surgical means. To paraphrase Mark Antony's funeral oration—the evils of operations live after them, but the good is interred with their bodies. The quack adroitly turns these facts to his advantage and plays upon this false interpretation to his own evil gain.

In the booklet of a notorious institute, which has a wide circulation, under the title of "The Use of the Knife," the following opening sentence is quoted: "The great majority of either cancers or tumors are of such a nature that the use of the knife simply retards their destructive work, driving them more deeply into the system and allowing them in time to return with renewed and increased vigor." How artfully such a statement covers the ill results of surgery. How skillfully it appeals, even to an intelligent person, who has had some friend or member of his family unsuccessfully operated upon. As he has witnessed the course subsequent to operation, there has been no apparent, or at most only temporary relief, when the metastatic deposit in the liver or other organs becomes evident, and with its loathsome train of wretchedness and misery it is not unreasonable that both the family and friends should become bitter pessimists. After painting the picture of this disease in a pseudo-scientific manner, which so appeals to the vanity and credulity of many laymen, the charlatan offers his simple, painless and positive cure.

In another booklet before me, which is carried by our postal system to thousands of homes, the seducive invitation to certain death is worded as follows: "Our specialists through years of experience in the treatment of cancerous and other growths have established the fact that cancer is a curable disease, and that our remedy is so sure and safe that we are able to give a written guarantee of permanent cure, a thing no other specialist can do."

Polemics against such quacks fall as the riven dart from an impregnable wall, for as public opinion now stands in open combat we can not command the voice of the people, and the legislative contest is, therefore, usually fruitless. Only by the gradual enlightenment of the public through popular channels of education, the potent agencies that the charlatan employs, will the desired end be reached, and even this course will be tedious and full of disappointments.

### HOW SHALL WE EDUCATE THE PUBLIC?

Hedged about by our ethics medical knowledpe has been in the past too much the property of a closed corporation. This is manifestly wrong, for how can we hope for the co-operation of the lay world unless we furnish them with an intelligent conception of our aims? Happily the turning point is behind us, for not only have many of the most important periodicals taken up educational campaigns in matters of public and personal health, but the American Medical Association has assumed this function and has constituted a Board of Public Instruction for the dissemination of all facts which may be utilized by the world at large.

In an educational campaign with regard to cancer we must necessarily steer a very guarded course, for to generate in the mind of the neurotic the fear of this disease, when it is not present, could only result in evil consequences. The word cancer carries with it such terror and so completely crushes hope that the enlightenment of women must be carefully attempted, and they must be assured that not every lump in the breast is cancer, nor every atypical menstrual flow an indication of malignant changes in the uterus, but that such deviations must at once have attention in order to prevent a more serious condition.

It is a well-known fact that the tubercular subject is always hopeful, while upon the contrary the cancerous patient is cast into the very slough of despond by the suggestion of his disease. Instruction, therefore, must be given with a view to merely enlightening the public concerning suspicious symptoms in order to secure their earlier attention. Happily in a majority of women with these symptoms, even in the cancerous age, no trace or suspicion of the disease will be found upon examination and only some trivial lesion will be the cause of the atypical bleeding. While therefore, the statement may be adhered to that the alarmist's viewpoint is, in general to be decried, in cases of cancer of the uterus there is little danger from this attitude, for if a fear of the disease has been aroused by suspicious signs, it may quickly be set aside or confirmed by an examination. There is not in this disease that long, anxious waiting for symptoms to unfold themselves to confirm the diagnosis. Soul-racking apprehension of impending evils can, therefore, be at once established or set at rest. If the diagnosis is positive, a precipitate operation should be urged; if, on the other hand, there is no cancer, the patient is all the happier and all the safer for this knowledge. Under these conditions she will not become morbidly introspective, as is so usual with the person harboring the secret fear of this disease. Now that the ignorant as well as the intelligent women are coming to learn that operation is the only hope held out for their cure, the very fact that a negative diagnosis is followed by the assurance that no operation, or at most only a trivial one. is required, at once allays all fears.

Lectures before women's clubs, and particularly the judicious talks of physicians to patients, are likely to be most effective. We must certainly in the end appeal, through popular channels of publication, to the lay world; but as in other medical subjects, such instruction must be supervised carefully to prevent the arrow going wide of its mark.

---

### THE PLACE OF THE X-RAY IN THE TREATMENT OF CANCER.

By John C. Price, M. D., Scranton.

In this paper I will use the term cancer as meaning all malignant growths and treat the subject under three principal heads: First, the superficial epithelial growth; second, the deep-seated epithelial growth; and third, sarcoma.

For the treatment of superficial epitheliomata of the skin, I believe that in x-ray we have the best remedy, and it has been my experience that when the treatment has been thorough, there is less likelihood of return than after any of the other forms of treatment. It has the advantage that there is no pain; no unnecessary removal or destruction of healthy tissue, and consequently a minimum of scarring, which is of particular importance where the lesion is located on the face.

The epitheliomata of mucous membranes are much more resistant to the x-ray, and therefore should never be primarily treated with it unless there is some strong contra-indication to an early and complete operation. It has been my experience in the few cases of this kind that I have (by my early enthusiasm, and later by necessity) treated, that in too large a percentage the treatment has proved unavailing, but I have had some good results where operation was contra-indicated, and therefore would urge the use of the x-ray in this class of cases following operation, or, if operation is absolutely out of the question, as a primary treatment.

In the treatment of the more deep-seated epithelial growths the primary treatment by x-ray should never be considered, unless as a palliative measure for inoperable cases. In these, if the tumor mass can be removed without too great an injury to the patient, I believe that it is always advisable to have it done, as the ray has less tissue to penetrate, which very much increases its efficiency; also, there is much less necrotic tissue to be absorbed or thrown off with the attending toxic effects to the patients.

That the x-ray does have a very decided beneficial effect in most of these cases, is established beyond question. It relieves suffering, and prolongs life under more bearable conditions than we can obtain without it. There are a few cases that the ray does not seem to affect, probably due to a difference in the type of cells in the growths. The greatest field of usefulness of the x-ray in this type of growth is in the treatment during and after operation. It is most important that the wound should be thoroughly rayed before it is closed. It is very evident that the small islands of malignant tissue that may be left are much more easily reached with the wound wide open, than after the skin flaps are closed, and if there are any particles of the growth left in the wound to be absorbed during healing, which may cause metastasis, they are much more easily affected at the time of operation than later when they have started new growth, perhaps, in the deeper tissues. The wound should be covered with several layers of wet gauze to prevent infection from particles of dust, and the raying should be very thorough with a medium or rather soft tube, twelve inches from the patient, excited by from one to two milliamperes of current, for from twenty-five to thirty minutes. Short exposures at such a time I consider worse than none, as they may stimulate rather than retard the growth of the diseased tissue. So far as I have been able to find out, this treatment does not interfere with the healing of the wound. The treatment should be kept up afterwards, using a Pfahler's sole-leather filter, and pushed as far as the patient's skin will permit. After fifteen or twenty exposures the patient should be allowed an interval of three to six months' rest if there is no sign of return, and then another course of treatment given.

In the treatment of these cases where the growth has returned, I believe, where possible, the nodules should be removed and a very thorough course of x-ray given. In this class of cases, and especially where there is metastasis to the deeper tissues, I consider that the use of Pfahler's sole-leather filter is of the greatest importance, as the exposures can be more than doubled without danger of seriously injuring the skin.

In the treatment of sarcoma the ray affords one of the best auxiliaries to surgery; where possible, of course, I believe in early and radical removal, but when the tumor is of large extent and can not be wholly removed, it should be vigorously rayed, and as soon as there are signs of breaking down, the diseased mass should be removed to prevent absorption of toxins and to allow the ray more free access to the deeper parts of the growth, and then the treatment should be vigorously pushed. In the treatment of sarcoma, surgery and the x-ray should go hand in hand more than in any other class of malignant growths.

The treatment of metastasis and recurrence should be conducted as that of the original growths. The technic that seems best in sarcoma is the use of an old, hard tube with a sole-leather filter giving long exposures, from twenty-five to forty minutes, the tube being about twelve inches from the skin, and excited by one milliampere of current.

It has been my experience in the treatment of the deep-seated malignant growths that the use of hard tubes and long exposures give the best results. I have found that the skin will stand long treatment with a hard tube without serious injury, while a soft tube soon gives trouble; and that often the growth of the diseased tissue is stimulated rather than inhibited by the shorter exposures.

As a summary, I would state that the x-ray is very effective in superficial epitheliomata of the skin; that, in the treatment of all other malignant growths, it should be used only as an auxiliary of surgery, or as a palliative in inoperable cases, and that any surgeon who operates on malignant cases and does not urge the use of the x-ray treatment when it is possible, is failing in his duty to the patient and to himself.

---

### THE X-RAY IN RELATION TO CARCINOMA OF THE BREAST.

By William S. Newcomet, M. D., Philadelphia.

There is not the least doubt that there exists in our community a large number of people who have a decided fear or aversion toward the medical profession, and for the substantiation of this fact it is only necessary to call attention to the opposition to vaccination. In this instance one can scarcely understand why it should be so, considering the universal good that has followed this procedure. On the other hand, when it comes to an operation for cancer perhaps the most sanguine of us may excuse those who do not follow our advice of "early and thorough removal of the disease," for there are few of us that care to undergo this very trying ordeal without some hesitation; however, this procrastination upon the part of the sufferer usually means a delay that in many instances gives the disease more time to become more widely scattered through the system, and in some to such an extent that recovery is a past issue.

Some may think these statements are too pessimistic, but there is not the least doubt that the general public are in total ignorance of how to protect themselves against malignancy. These rather startling facts were evident upon reviewing the records of the American Oncologic Hospital. In a list of 117 cases of carcinoma of the breast, forty-two were regarded as neglected when they were examined for admission. This means that at that time there was well-marked extra-glandular involvement, and the case was considered hopeless. Of these, twenty-two simply allowed the disease to follow an uninterrupted course; in thirteen, plasters or ointments were used; five used medicines, hops being the remedy in two. Another used the galvanic battery while two others used radium products. In some instances medical advice was never sought while in others it was disregarded; on the other hand, some of the remedies were prescribed by physicians. In general the disease was recognized and it was allowed to progress with a perfect understanding as to its nature.

A comparative study of some of these neglected cases would be most interesting. To some extent, they explain the results after different operative procedures, and they show the wide variation of malignancy, where it is allowed to follow its own uninterrupted course, from the very rapid case (that of a few months' duration, scattering from a local spot to almost the whole body) to the one in which it practically never causes death and, in matter of fact, as the patient advances in years and the tissues of the body become shrunken it too starts to shrivel just the same as the surrounding elements upon which it is embedded. These last mentioned cases are not so uncommon, but the spontaneous disappearance of carcinoma is rare and extremely doubtful.

It seems from the more recent study of this disease that metastasis occurs much earlier than is usually supposed, and this seems especially true of carcinoma of the breast. There is not the least doubt that the axillary glands are involved in more instances than is usually accredited, and the routine examination of the glands microscopically should be universally adopted, for if these glands have been invaded, it is likely that the cervical, bronchial and other chains are also involved.

These are the cases where early x-ray treatment may prove to be of the greatest value, for the same rule holds good here as it does in early operation, and just as many failures arise from the treatment being used too late. If the carcinomatous infection is so slight that it requires the microscope to demonstrate its presence, there is not the least doubt that there is a greater chance for disappearance than when it is perfectly evident to a superficial examination. An instance of the early dissemination of the disease is well illustrated by the short synopsis of the following case:

Miss S., aged forty-three years, white, about nine months ago noticed a small lump in the right breast. It did not change in size and it was not until a friend developed serious trouble from a like lump that attention was given to it. Previous history was unimportant. At the time of operation this mass was about the size of a small bean and upon examination by Dr. John Swann, it was found to be a fibro-adenoma that was undergoing carcinomatous degeneration, and the cells had already broken through the basement membrane. Dr. A. Hewson then performed a complete operation and these tissues were examined by Dr. James Kelly; he found metastasis upon the posterior aspect of the pectoralis major, and also in the axillary glands. The interesting points in this case were the small tumor, the wide dissemination of the disease, and again the patient was only induced to seek medical aid by the alarm caused by a friend having a like tumor that had undergone some simple absorption treatment.

Occasionally one will see some remarkable instances of advanced disease yielding to the power of these rays; however, it is not usual, for the best results are to be found by prompt and energetic treatment and here alone is the only hope of removing the disease. Valuable time is often lost between a series of operations, for when the disease once recurs it is very likely to occur again, and if the x-ray has not been employed after the primary operation as a prophylactic, it should immediately follow the second, and a wide area should be exposed.

In some instances when recurrence happens, the disease is too widespread to permit of any further operative procedure. These cases are not as a rule very hopeful ones, but the patients sometimes recover after vigorous treatment, and the following case, referred by Dr. Wharton, is a typical example.

Miss XX., aged about forty years. On July 26, 1906, right breast contained a mass about the size of an egg and the axilla was also involved. The whole area was cleared of disease, and at that time the case was regarded as doubtful. On May 10, 1907, on the skin over the chest, over the shoulder and extending down the back to the body of the scapula, were a number of red spots which were elevated. The largest were about half an inch in diameter; they were larger and more numerous along the line of incision. The arm was not involved. The usual method of applying the x-ray was employed and two months later there was not the least trace of the disease. On November 27, 1908, the patient again returned with several doubtful spots upon the chest and a few more treatments were given when they again disappeared. In May, 1909, she again called and stated that she wished to spend the summer in Europe if her condition would permit, and at that time there was not the least evidence of the disease and it did not seem justifiable to detain her from her voyage. The number of treatments given was thirty.

However, while this case promised so little in the beginning from the widespread distribution of the disease, it yielded easily to treatment for the reason that it was promptly given when recurrence was discovered.

In contrast to the case just mentioned is the following, neglected from the start, and while these will often do well locally, other processes at distant points usually deplete the system, and cause a fatal issue:

Mrs. 776, white, fifty-two years of age, was well nourished; previous history was good. In her forty-ninth year she injured her breast; two years afterwards she noticed a lump but paid little attention to it, until a year later when it began to cause pain. In July, 1908, she applied for admission to the American Oncologic Hospital and at that time, upon examination, the whole breast was involved, the skin covering it was red and in one spot was ulcerated; the axilla was also involved. On July 7, Dr. Samuel McClary removed the breast and dissected out the axilla. The operation was very extensive and the flaps were not sufficient to cover the wound and left a triangular area uncovered. The patient developed pneumonia after her operation and this delayed her recovery. On account of the advanced condition of the malignant disease it was decided to use the x-ray as soon as her condition would permit. This, however, was delayed until August 29, and at that time there was considerable return of the disease, especially along the line of the incision and about the stitch holes; the triangle measured about three inches on each side. This recurrence improved under treatment but new areas outside the limits of the ray were constantly arising; it gradually involved the deeper glands until the patient died of exhaustion, May 29, 1909.

The synopsis of the foregoing case illustrates the marked effect of the x-ray upon carcinoma locally. It shows that the disease was to a great extent suppressed; it proves that if it is possible to discover the disease it is possible to combat it, but where it has made such tremendous inroads upon the system improvement is only temporary and the patient usually succumbs to deeper glandular involvement. Many of the cases that come before us are of this class and often patients have had as many as five or six local operations.

A more uncommon type is where the x-ray has literally no effect; in fact, it is supposed by some to do harm. These are usually very late cases with edema of the surrounding parts, and often with extensive ulcerations. It seems needless to expect such cases to react favorably; under these circumstances with the tissues in such condition they receive very little nourishment, ulceration is favored, and all these certainly prevent metamorphosis. Take the same conditions in a burn and the ulceration here runs a most protracted course under far better circumstances. Certainly the disease, and the individual, must be considered. There is a difference in resistance; at present the cause is obscure, and at the same time the idiosyncrasy to the x-ray must be remembered, but this is not so common as it was formerly believed, but with all due consideration of these facts in the immense majority of instances unfavorable reaction is due to poor nourishment of the surrounding tissues.

The following distressing case illustrates these points:

Mrs. XXX., aged forty-nine, white, came under observation, February 24, 1903. At that time there was a mass in the left breast about the size of a hickory nut; it was painful and the skin over it was reddened. It was first noticed about three years ago. Operation was advised but refused. On November 7, 1907, again she came under observation. During the interim she used some local applications prescribed by a physician, with no benefit whatever. Examination at this date showed that the left breast was greatly enlarged, and attached to the pectoral muscles there was an ulcerated area two and a quarter by four and three quarters inches which was discharging foul pus and the edges bled upon the slightest manipulation. A mass in the axilla was about the size of a goose egg but no glands could be felt in the neck. At this time she consented to an operation, which was performed by Dr. A. Hewson on November 12, 1907. The patient recovered promptly and on November 24 x-ray treatment was begun. For a time the case progressed favorably but in the early part of February the arm started to swell. It was decided to push the x-ray but by the middle of the month there was considerable reaction and it was necessary to stop treatment. The arm continued to swell and there was invasion of the disease in and about the old area; x-ray treatment was again given but it failed to hold the disease in check and it also set up considerable reaction. Trypsin and amylopsin were employed locally and hypodermically with no effect. The disease progressed with great rapidity until the end.

These cases just mentioned represent the usual types that come before us for treatment and as long as we know absolutely nothing as to the caues of this frightful malady we must to a great extent deal with empiricism. It has been clearly demonstrated that the x-rays are of value in certain forms of malignancy. Upon this point there can be no argument, but there still exists a considerable doubt as to when and in what forms of this disease the agent should be employed, for in some instances, by some observers, it has been claimed that it was even harmful. This is by no means impossible, for under some circumstances drugs that are known to be specifics in certain diseases will at times act to the contrary, and do more to disarrange the system than to assist in repair. However, it being granted true or untrue that the x-ray has done harm in the treatment of cancer, it must be conceded that these cases are very few in comparison with those that have been benefitted. Then, again, in these unfavorable cases we must ask if the x-ray was properly applied, and here no censure is intended. It must be remembered that this is a new agent and its many forms of application are not thoroughly understood. At the present time it is difficult to state the actual worth of the x-ray as a prophylactic immediately after a primary operation. To arrive at any conclusions as to its usefulness will require years of observation. Under the present operative conditions many cases escape further development of the disease and others only recur after a lapse of many years, often five or ten. The question, on the other hand, is to determine the extent of the disease. In other words, is the disease confined within its original capsule at the time of operation or has it invaded other structures and to what extent? This is a question for the pathologist alone and upon his report should depend the character of the treatment. However, if this still remains an open question, there can be no objection to the adoption of a rule that, when the axillary glands upon microscopical examination are found to be involved by the disease at the time of the primary operation, the whole field should be carefully treated by the x-ray. If recurrence should then happen, vigorous treatment should be given. In cases, where the disease recurs after extensive operation, that have not received x-ray treatment as a prophylactic, it should be immediately instituted in conjunction with any other operative procedure that may be required. But until we know the exact nature of cancer it is impossible to give any exact rule that will apply to the general run of cases and until that time it must be a matter of judgment for the individual case.

